# Free Medical Schools

**Published:** 1850-01

**Authors:** 


### Part 4.—Editorial.

### ARTICLE 1.

### FREE MEDICAL SCHOOLS.

Messrs. Editors:—Under the above caption, the Editor of the *Western Lancet* devotes two or three pages of his November number, to a review of my recent introductory address in Rush Medical College. In doing so, our respected cotemporary has repeated one or two errors, which may be worthy of a passing notice. After stating with tolerable correctness, the principal topics discussed in the address, and representing the "inordinate expenses which the present system imposes on the medical student," as the leading subject discussed; he says:—"So far as we can determine, from the language of the Lecture itself, the author is advocating a free system of medical teaching,—that is, the teachers reward shall be the *glory* of imparting knowledge 'free gratis for nothing.'" The first part of this sentence is correct. The address does advocate a system of Free Medical Schools—*free*, in the same sense as advocated by Dr. Alex. H. Stevens, Ex-President of the American Medical Association, in an elaborate and exceedingly interesting address before the New York State Medical Society; and which attracted so much attention that the Legislature of that State ordered the printing of a large edition for the use of its own members. Free too, in the same sense as that adopted by the Regents of the University of Michigan, who, in establishing the Medical Department of their State University, have made the the *only* charge on the medical student, a matriculation fee of *ten dollars.* But the latter part of the above quotation has not an equal claim to truth.— The address no where pretends that "the teacher's reward shall be the glory of imparting knowledge *free gratis for nothing*;" but on the contrary devotes no inconsiderable space to well founded considerations, showing it to be alike the duty and interest of every State to "so *endow* its Medical School or Schools, that the *Lecture Fees* may be *entirely* abolished."—And until this is done it advocates such a reduction of the present high rates of charging as will bring them more nearly in correspondence with the actual pecuniary resources of a large majority of those who engage in the study of Medicine. It further claims that such a reduction of the fees, or their entire abolition, would induce the student to be in less haste to terminate the period of his pupilage, and enable him to attend a greater number of courses of Lectures, and greatly enhance the amount of his medical attainments before he entered upon the practical duties of his profession. And in the same proportion, of course, would the standard of attainments throughout the profession become elevated.

Nor is this all, for the address claims, and that truly, that such a change, by removing the only foundation on which all purely country Medical Schools have rested for their patronagee, viz: their *cheapness*, would rapidly concentrate the whole number of students in schools so located as to make accessible to their classes well regulated and well filled *hospitals*, duly supplied with good clinical teachers; advantages certainly no less important to the student than the mere oral instruction of the lecture room. Now, if it be desirable that the student should attend several sessions of a medical school before graduation, and if it be further desirable that he should attend such a school as can afford him true clinical instruction, not only in such chronic diseases and minor surgical operations as can be presented in a College Clinique, but also in all the graver forms of disease as they may be seen and illustrated in well sustained hospitals, then it is neither *visionary*, *ideal*, nor *utopian*, to advocate such a system or rate of charges as will ensure not only the attendance of students on such schools, but also induce them to attend the longest possible period.—And it can require no great stretch of either reason or imagination, to see clearly that just in proportion as this object is accomplished, in the same proportion will the cause of sound medical education be advanced. What has been the experience of our friends at Cincinnati? During more than twenty years they have been struggling to sustain and build up the Ohio Medical College, located in that beautiful "Queen City of the West," with ample material for hospitals, and every collateral aid that a Medical College needs.

During the time they have had some thousands of dollars appropriated to their aid by the Legislature of their State,—have numbered among their teachers some of the ablest in America; and have uniformily enjoyed the benefit of high charges, i. e. $15 00 per ticket, or from $90 00 to $105 00 for a full course. The result is, after this protracted enjoyment of all these advantages, the collection of an annual class of no more than 125 or 150 actual students; while in half the time two other schools have sprung up in the same State, and with about half the rate of charges, have secured an annual class of nearly double the number of students; and what is far worse, two *quack* schools have sprung up within sight of their own doors, well nigh rivalling themselves in prosperity. Does not such a result as this, indicate that there has been something in the management of the Ohio Medical College, which smells strongly of "*closet cogitations* and *speculative notions;*" something "*ideal,*" or at least not very nicely adapted to the *actual* condition of things, and therefore not quite as "*practicable*" as desirable?

But suppose the Cincinnati faculty had made the important discovery announced in the last paragraph of the article in the *Lancet*, viz: "that two hundred students at five dollars a ticket, would pay just as much as one hundred at ten dollars," ten years since, and had immediately adopted it as a rule in practice, thereby reducing their fees from $15 00 to

$5 per ticket, does any one doubt tnat they would now be lecturing to a class of 400 or 500, instead of 150 students? And would there be any difficulty in determining which number would be most profitable to the faculty? The only reply which the editor of the *Lancet* has made to the arguments and positions presented in the address, is contained in the following paragraph, viz: "His proposition is, that Medical Schools should all be free; very well, let the principle be carried out. There are, no doubt, some who are not only unable to pay their tuition fees, but also their clothing, travelling expenses, board, &c. Now it is certainly necessary that provision be made for all these expenses; and as the author contemplates lengthening the sessions, it will be necessary to make tolerably ample provisions, for we have no doubt that such a school would be extensively patronized. But further than this, we would most respectfully suggest, that, after the *doctor* has been thus gratuitously educated, his bills should all be paid by the government," &c.

We suppose our logical cotemporary, in carrying out *the principle* in the same direction, would say to the State of New York, inasmuch as she has provided a system of *free* schools for the education of all her children, therefore she ought to provide them all with food and clothing, and see that their debts are paid. There would be just as much good sense in the one case as in the other. But let us see how the *principle* would look carried a little way in the opposite direction. The *Lancet* assumes that cheapening medical education degrades and injures the profession, and of course the converse also, that increasing its expensiveness, elevates and improves it. If this principle be true, then every medical school is in duty ou nd to charge not the *trifling* sum of $15 00 per ticket, but $30 00 at least; and the community where such schools are located, instead of providing cheap boarding houses for the accommodation of students, should always exact of them double price for every thing in relation to board and clothing.

We had been long laboring under the impression that the character and usefulness of any profession depended almost entirely on the degree of intelligence and skill of its members; and this again depended mainly on the facilities afforded for acquiring knowledge and skill pertaining to the practical duties of such profession. We had supposed that the *free* medical schools of Paris, had been chiefly instrumental in making that city the great modern emporium of medical science; while a tenacious adherence to ancient usages and high charges had sunk the formerly famous schools of Edinburgh into comparative oblivion. In a word, we had supposed it a universally admitted axiom, that the surest way to advance the character and interests of any science, art, or profession, was to increase the facilities for acquiring a thorough knowledge of such art or profession in all its scientific and practical details.

According to the doctrines of the *Lancet*, however, these are all wild delusions; mere '*closet cogitations* and speculative notions;" a mistaking of the *ideal* for the *real;* while the true mode of elevating the medical profession, is to hedge up the pathway to knowledge, and tax the student's *pockets* instead of his *brains*.

Verily, France must change her policy, and the schools of Paris must close their doors to every man who has not $100, or $200 to pay for each course of lectures, or the profession in that beautiful country will be speedily ruined. The only objection to free or cheap medical schools which bears the semblance of plausibility, is, that they would induce a greater number to engage in the study of medicine, and consequently add to the crowded state of the profession and all the evils resulting therefrom. But however plausible this objection may appear to the unreflecting mind, its fallacy is clearly demonstrated by experience, by analogy, and by the present actual condition of the country.

In regard to experience, we have already alluded to the marked influence exerted by the establishment of free medical schools in Paris, and on this we might rest until our opponents succeeded in demonstrating that the making of Paris the head and centre of Medical Science, had *degraded* the profession in that city and country.

But let us look a little to the experience of the profession in our own country. Previous to 1765, there was no medical school in America; and all who attended medical lectures, or became graduates, were compelled to cross the Atlantic, and spend the requisite time at the expensive schools of Edinburgh or London. This, at least, made medical education as *expensive* as the most fastidious could desire; and according to the advocates of high charges, ought to have placed the profession in the colonies on the highest eminence, as regards its honorable standing and scientific attainments. But what was its actual, its *real* condition? Dr. Nicholas Romaine, in his annual address before the Medical Society of the State of New York, in 1811, says: "It would be painful to intrude on your notice the *humble* condition of medicine, which seems to have existed for more than a century after the first settlement of this (New York) State. It could only consist of a statement of the *arts* and *intrigues* by which the practitioners of Physic succeeded in advancing their *private interests* and professional emoluments, &c." And we are told by the Independent Reflector, an able paper published in the city of New York in 1753, "that it (the city of N.Y. then containing about 10,000 inhabitants) could boast of more than *forty* gentlemen of the faculty, the greater part of whom were mere pretenders to a profession of which they were entirely ignorant."

Again, we find a law passed by the Massachusetts Colony in 1659, forbiding "*Chirurgeons, Midwives, Physicians, or others*; to exercise or put forth any act contrary to the known rules of art, in each *Mystery* and occupation, or exercise any force or violence, or cruelty, upon or towards the body of any, whether young or old, (not even in the most desperate cases) without the advice and consent of such as are skillful in the same art, (if any such may be had) or at least, of the wisest and gravest there present, and the consent of the patient or patients, if they be mentis compotes, much less contrary to such advice and consent, &c." These extracts, selected from a great number that might be made, are abundantly sufficient to prove two things, viz: First, that the profession of medicine was, at that time, in an exceedingly low and ignorant state; and second, there was no scarcity of practitioners, (such as they were;) for "*more than forty*," in one city of 10,000 inhabitants, is putting them quite as thick as they are to be found at the present day, even in the vicinity of the *cheapest* medical schools. Here, then, we have as expensive a system of medical education as any could desire, and as a direct consequence, an abundance of practitioners, of whom forty-five out of every fifty were either priests or the merest charlatans.

But time passes on, and medical schools become established and multiplied, throughout the whole country, and in the same proportion the facilities for acquiring a medical education become increased, and its expensiveness diminished.--- Does the relative number of practitioners, however, increase in the same proportion! Not at all. But a far larger proportion of those who do engage in the profession, are induced to resort to the schools and gain at least respectable attainments before commencing to practice. Until it is satisfactorily shown that there is now a greater number of practitioners in proportion to the number of inhabitants, than there was of "Chirurgeons, Midwives and Physicians" in 1753, we shall assume that experience demonstrates that increasing the facilities for acquiring medical knowledge and diminishing its expensivenes, has not, in times past, either degraded the profession, or rendered it more crowded. We have said that the fallacy of the objection was shown by analogy also.

Thus the legal profession in this country, has ever been perfectly free to all who were able to furnish themselves with food and clothing during the required period of study, and could pass the proper examination at its close. No expensive collegiate courses of legal instruction have been interposed to limit the number of law students, and yet we are not aware that this cheap system of legal education has either lessened the dignity or character of that profession, or so crowded its ranks as to produce a ruinous competition in regard to the rate of charges.

The truth is, that ever since mankind have been subject to disease and injury, a class of men have existed whose business it is to endeavor to repair injuries and heal diseases; and their numbers have ever been proportionate to the population and sanitary condition of the community where they live. In the heathen and uneducated tribes of the human family, the functions of Physician and Priest are generally united in the same class of persons, while surgery dwindles to the lowest of mechanical employments. In such countries, there are no facilities for any thing worthy of the name of medical education, yet there is no proof but their *Medicine Men*, *Midwives*, &c., are as *numerous*, in proportion to the population, as in the most enlightened countries. But in the same proportion as communities become enlightened, and the facilities for acquiring true medical knowledge are increased, in the same proportion does the profession become distinct and separate from other employments, respectable in its character, and enlightened in the discharge of its duties. This is a truth which can neither be gainsayed nor disproved. Thus, in New-England and New-York, so late as 1812, medical schools were few, being confined to Boston, New-Haven, and New-York city; and three fourths of the practitioners had never graduated, and more than half had never attended medical lectures at all. Now it is comparatively very rare that a man commences regular practice in any of these States without a regular College Diploma. But in the West, and much of the South, we still find a very large proportion of those who engage in practice, doing so without ever going to a Medical College, or at most, attending but one course of lectures. In the East, their fees are moderate, the schools easy of access, and the rate of interest on borrowed money low. While in the West and South, until within the last few years, the colleges were more remote, the lecture fees one third higher, and the rate of interest on money increased one half. In short the facilities for acquiring medical knowledge, have been, for many years, one-third greater in New-York and New-England, than in the West and South. The actual result is, not an absolute increase of the number of physicians in proportion to the population, in the first named section, more than in the last; but the submission of a far larger proportion of them to a full course of study and graduation.

The great fundamental truth is, that the number of those who engage in the practice of medicine, as in every other art, or calling, or trade, is regulated wholly by the law of *demand* and *supply*. And just in proportion as you throw obstacles in the way of acquiring medical knowledge by charging high tuition fees or otherwise, you increase the proportion of those who practice without proper qualifications, and *vice versa*, without at all affecting the absolute number, Unless indeed, you assume that the greater the proportion of half qualified ones, the greater will be the absolute number required to do the business—a conclusion by no means without some foundation.

*December* 19, 1849. N. S. D.

